# A modified lung ultrasound score to evaluate short-term clinical outcomes of bronchopulmonary dysplasia

**DOI:** 10.1186/s12890-022-01885-4

**Published:** 2022-03-19

**Authors:** Ying-Hua Sun, Yang Du, Jie-Ru Shen, Dan-Yang Ai, Xiang-Yuan Huang, Si-Hao Diao, Sam Bill Lin, Rong Zhang, Lin Yuan, Yi-Pei Yang, Li-Li He, Xiao-Jiao Qin, Jian-Guo Zhou, Chao Chen

**Affiliations:** 1 Department of Neonatology, Children’s Hospital of Fudan University, National Children’s Medical Center, No. 399 Wanyuan Road, Minhang District, Shanghai, 201102 China; 2 Department of Ultrasound, Children’s Hospital of Fudan University, National Children’s Medical Center, Shanghai, China; 3 Department of Pediatrics, The Children’s Hospital, Zhejiang University School of Medicine, National Clinical Research Center for Child Health, Hangzhou, Zhejiang China; 4 Department of Clinical Epidemiology, Children’s Hospital of Fudan University, National Children’s Medical Center, Shanghai, China; 5 Saw Swee Hock School of Public Health, National University of Singapore, Singapore, Singapore

**Keywords:** Lung ultrasound, Bronchopulmonary dysplasia, Premature

## Abstract

**Background:**

Lung ultrasound (LUS) is a useful tool for assessing the severity of lung disease, without radiation exposure. However, there is little data on the practicality of LUS in assessing the severity of bronchopulmonary dysplasia (BPD) and evaluating short-term clinical outcomes. We adapted a LUS score to evaluate BPD severity and assess the reliability of mLUS score correlated with short-term clinical outcomes.

**Methods:**

Prospective diagnostic accuracy study was designed to enroll preterm infants with gestational age < 34 weeks. Lung ultrasonography was performed at 36 weeks postmenstrual age. The diagnostic and predictive values of new modified lung ultrasound (mLUS) scores based on eight standard sections were compared with classic lung ultrasound (cLUS) scores.

**Results:**

A total of 128 infants were enrolled in this cohort, including 30 without BPD; 31 with mild BPD; 23 with moderate BPD and 44 with severe BPD. The mLUS score was significantly correlated with the short-term clinical outcomes, superior to cLUS score. The mLUS score well correlated with moderate and severe BPD (AUC = 0.813, 95% CI 0.739–0.888) and severe BPD (AUC = 0.801, 95% CI 0.728–0.875), which were superior to cLUS score. The ROC analysis of mLUS score to evaluate the other short-term outcomes also showed significant superiority to cLUS score. The optimal cutoff points for mLUS score were 14 for moderate and severe BPD and 16 for severe BPD.

**Conclusions:**

The mLUS score correlates significantly with short-term clinical outcomes and well evaluates these outcomes in preterm infants.

**Supplementary Information:**

The online version contains supplementary material available at 10.1186/s12890-022-01885-4.

## Background

Bronchopulmonary dysplasia (BPD) is a severe pulmonary complication of prematurity, and the leading cause of post-neonatal death in extremely preterm infants in neonatal intensive care units (NICUs) [1, 2]. The current National Institute of Child Health and Human Development (NICHD) consensus definition of BPD is based on oxygen requirement and clinically assessed need for supplementary respiratory support at 28 days postnatal age and 36 weeks postmenstrual age (PMA) [3]. Although the criteria provide a much-needed diagnostic framework, it’s not designed to predict future pulmonary disease outcomes. Indeed, there are few prognostic markers with sufficient reliable clinical ability to evaluate the level and duration of respiratory support.

Lung ultrasound (LUS), as a point-of-care diagnostic tool without radiation for critically ill patients [4, 5], has been used to describe typical neonatal respiratory diseases, such as meconium aspiration syndrome [6], respiratory distress syndrome [7], transient tachypnea of neonates [8], pneumothorax [9] and neonatal pneumonia [10]. In addition, LUS can accurately guide surfactant replacement therapy for neonatal respiratory distress syndrome [11, 12]. Based on these evidences, LUS has been recommended in the European point-of-care ultrasound guidelines for neonatal and pediatric intensive care [13]. However, only a few studies have investigated the utility of LUS in the diagnosis of BPD.

A classic six-chest area lung ultrasound score (cLUS) has been proved in accurately predicting the diagnosis of BPD [14–19]. In addition, previous studies have also shown that trans-hepatic and trans-spleen lung base ultrasound also significantly correlated with the development of BPD [20, 21]. Thus, it seems logical to combine the six-chest area and two-lung base ultrasound into a modified lung ultrasound (mLUS) score which is potentially more accurate to evaluate BPD severity.

In this study, we aimed to adapt a LUS score to evaluate BPD severity and assess the reliability of mLUS score in evaluating short-term clinical outcomes. We hypothesized that mLUS score correlates well with short-term clinical outcomes and has better diagnostic values in preterm infants compared with cLUS.

## Methods

### Study subjects

We designed a prospective diagnostic accuracy study in accordance with the Standards for the Reporting of Diagnostic Accuracy Studies (STARD) guidelines [22]. Patients admitted to the intensive care unit (NICU) of Children's Hospital of Fudan University from January 2017 to December 2019 were recruited for this study. Consecutive preterm infants born less than 34 weeks of gestation age and admitted to the NICU before 28 days of life were eligible for the cohort. Exclusion criteria were:(1) clinical evidence of severe congenital heart disease; (2) major congenital abnormality; (3) chromosome abnormalities; (4) suspected muscular dystrophy or neurological disease that may affect lung development; (5) lack of parental consent and (6) death or transfer before 36 weeks of the PMA.

The study protocol was approved by Children’s Hospital of Fudan University Institutional Review Board and informed consent was obtained from the parents (IRB No. 2015-194).

In our institution, we used standardized respiratory care and BPD procedures for management. This protocol was derived from the American Academy of Pediatrics guidelines [23], European guidelines [24] and Chinese guidelines [25], and has not changed over the course of the study (See Additional file 1: Appendix 1). BPD status was assessed at 28 days postnatal using the modified NICHD/NHLBI definition and severity was assessed at 36 weeks of PMA or 56 days of life [3, 26]. For more information, see Additional file 1: Table S1. Standardized systemic steroid therapy would generally be initiated when invasive positive pressure ventilation was required for more than 10–14 days [27]. This indicates a high risk of severe pulmonary disease and severe BPD.

### Lung ultrasound protocol

Lung ultrasonography was performed with the Voluson TM S8 Pro ultrasound device (GE Health Care, Milwaukee, Wisconsin, USA), a 9L-RS linear probe and an 8C-RS convex probe, with a maximum depth of between 50- and 80- mm. Lung ultrasound was performed by one well-trained sonologist at 36 weeks PMA. Lung ultrasound was performed when the infant was quietly lying supine in the incubator. Images were stored in a separate server not accessible by the clinical teams. These images were not included in the patients’ files and masked to other clinicians.

**Fig. 1:**
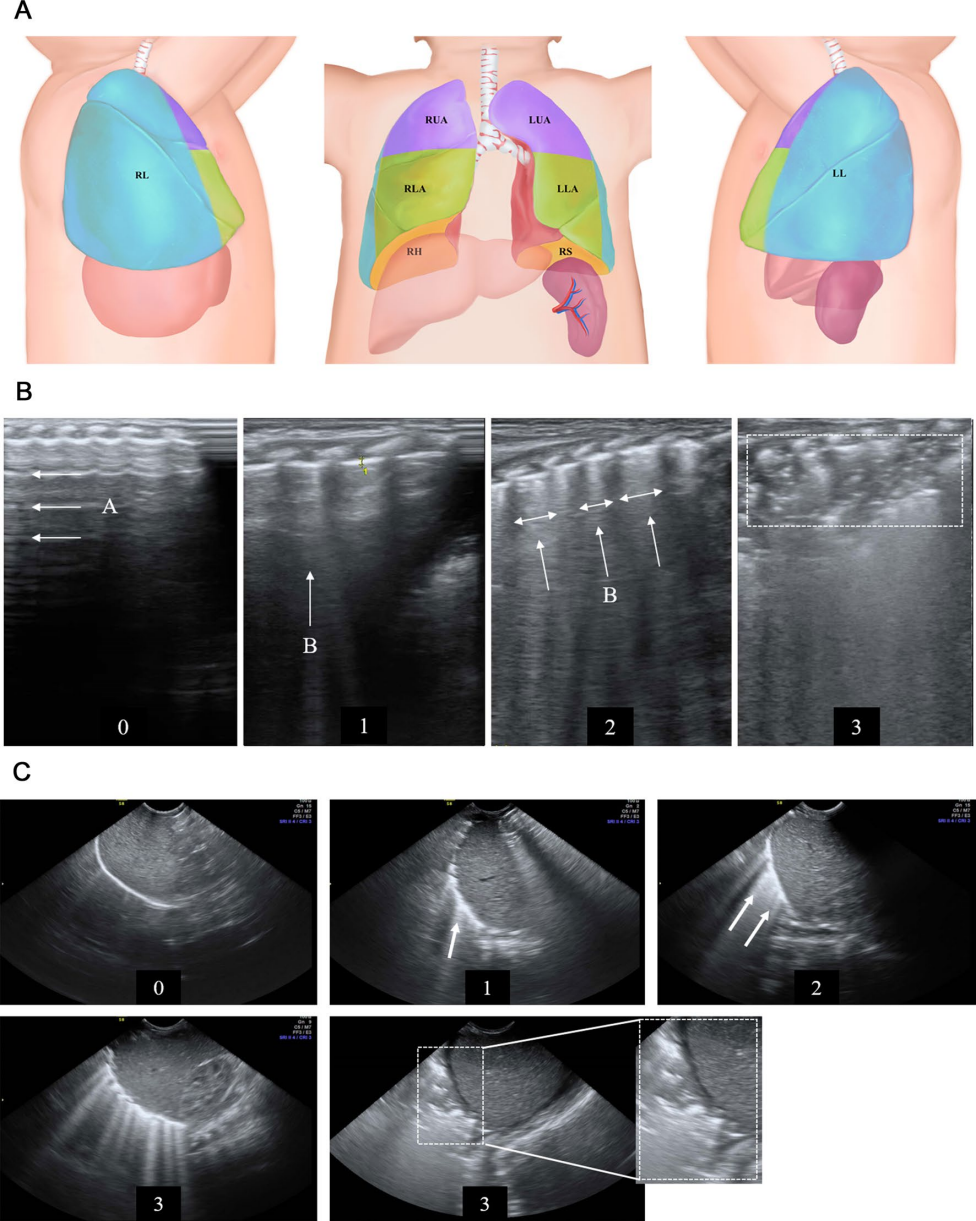
Description of the modified lung ultrasound score. Each lung has been divided into 4 areas. For each area, a 0- to 3- score was given, total score ranging from 0 to 24. Score values correspond to the different lung ultrasound semiology patterns, as shown in the middle and lower parts of the figure. RUA: right upper anterior; RLA: right lower anterior; RL: right lateral; RH: retrohepatic; LUA: left upper anterior; LLA: left lower anterior; LL: left lateral; RS: retrosplenic

Our modified lung ultrasound (mLUS) score was extended from the classic lung ultrasound (cLUS) score for newborns, as described in previous studies [11, 12, 16]. For classic lung ultrasound, each lung was divided into 3 areas (upper anterior, lower anterior, and lateral) and examined using linear probes through transverse and longitudinal scans. Each chest area can be marked on a scale of 0 to 3 (overall scale: 0 to 18). In our modified lung ultrasound, sagittal scans of the liver and spleen were also performed from below the lower end of the ribs using a convex probe. An up-scan was performed to visualize the lung base, and a score of 0 to 3 was given for each side. The mLUS score was calculated from six chest areas of cLUS examination and two additional retrodiaphragmatic areas (4 per side, total score ranging from 0 to 24). The details are described in Fig. 1 and Additional file 1: Appendix 2.

Images were recorded for each chest area and were reanalyzed by one investigator with expertise in lung ultrasound and blinded to the clinical condition. That was the best way to mask the clinical conditions to the investigators performing lung ultrasound and the lung ultrasound results to other clinicians. The images of the first 30 participants were used to calculate the inter‐observer variability for image interpretation by a second independent blinded investigator. Interobserver variability can be assessed using the intra-subject coefficient of variation. The intra-subject coefficient of variation is calculated as the ratio of the standard deviation of measurement differences to the mean of all measurements, which provided a scale-free and unit-free variation estimate between two independent investigators.

### Outcomes

Short-term clinical outcomes were as follows: (1) NICHD/NHLBI BPD severity (none, mild, moderate or severe); (2) respiratory support at 40 weeks PMA (none, oxygen/flow or ventilator/deceased); (3) oxygen requirement at discharge (none or oxygen/deceased); (4) postnatal systemic steroids treatment; (5) duration of mechanical ventilation; (6) duration of supplemental oxygen and (7) length of hospital stay. Study outcomes were as follows: (1) describing the correlation between mLUS score and short-term clinical outcomes; (2) evaluating the short-term clinical outcomes using the mLUS score, and comparing the ability to evaluate the short-term clinical outcomes with cLUS score.

### Statistical analysis

Continuous variables were presented as means ± SD or median (interquartile range) as appropriate. Categorical variables were presented as percentages. Comparative analysis was performed using Wilcoxon rank-sum test. Spearman coefficient (r) and regression coefficient (β, adjusted for gestational age) were used to assess correlations between mLUS/cLUS score and outcomes. Receiver operating characteristic (ROC) analysis was used to evaluate the abilities of mLUS/cLUS score to evaluate short-term outcomes [28]. Areas under the curves (AUCs) were reported and compared using the DeLong method [29]. The best cutoff values of mLUS score to differentiate (1) moderate and severe BPD and (2) only severe BPD were defined by Youden’s index calculation.

Sample size was calculated for primary outcomes as follows. To differentiate moderate and severe BPD, we targeted an area under curve (AUC) of at least 0.7 and considering as null hypothesis the prediction by chance (AUC = 0.5) and a positive/negative (no and mild BPD/moderate and severe BPD) case ratio of 1. Considering these data, 82 participants would have been needed with α = 0.05 and β = 0.1. Given the probability that some participants would be lost to follow-up, we enlarged the recruitment to ensure similar numbers of positive and negative cases in the cohort.

## Results

Between January 2017 and December 2019, 166 eligible preterm babies were admitted to the NICU, of which 38 infants excluded and 128 infants were finally enrolled in the cohort (See Additional file 1: Figure S1). The mean birth weight for the cohort was 1218 ± 392 g and the mean gestational age (GA) was 28.8 ± 2.3 weeks. Of these, 72 (56.0%) were male, and only 38 (29.7%) received complete course of antenatal steroids. Table 1 reveals the characteristics of all infants with BPD severity: the increase in BPD severity was inversely proportional to gestational age, birth weight, and Apgar 5-min score. As the severity of BPD increased, the percentage of infants who received PDA medical treatment increased. Infants with increasing severity of BPD also had more probability to receive postnatal systemic steroids, respiratory support at 40 weeks PMA, oxygen requirement at discharge, longer time of mechanical ventilation and hospital stay.

**Table 1:** Demographic characteristics of the study population

Characteristic	Non-BPD	Mild BPD	Moderate BPD	Severe BPD	*P* value
N	30	31	23	44	
Gestational age, weeks	31.5 ± 1.5	28.0 ± 1.5	28.8 ± 1.6	27.6 ± 1.9	< 0.001
Birthweight, g	1688 ± 374	1106 ± 233	1132 ± 259	1021 ± 285	< 0.001
Small for GA	2 (6.7%)	3 (9.7%)	2 (8.7%)	5 (11.4%)	0.92
Male sex	15 (50.0%)	17 (54.8%)	13 (56.5%)	27 (61.4%)	0.81
Cesarean section delivery	17 (56.7%)	14 (45.2%)	12 (52.2%)	16 (36.4%)	0.34
Complete antenatal corticosteroids	8 (26.7%)	10 (32.3%)	7 (30.4%)	13 (30.4%)	0.23
Apgar 5-min score	8.9 ± 1.1	8.4 ± 1.3	7.6 ± 2.0	7.8 ± 1.7	0.01
PDA medical treatment	1 (3.3%)	10 (32.3%)	11 (47.8%)	22 (50.0%)	< 0.001
IVH grade > 2	4 (13.3%)	3 (9.7%)	6 (26.1%)	8 (18.2%)	0.41
NEC grade ≥ 2	0 (0)	3 (9.7%)	3 (13.0%)	3 (6.8%)	0.28
ROP grade > 2	0 (0)	0 (0)	2 (8.7%)	5 (11.4%)	0.07
Sepsis, clinical or culture proven	11 (36.7%)	19 (61.3%)	13 (56.5%)	20 (45.5%)	0.22
Postnatal systemic steroids	0 (0)	0 (0)	1 (4.3%)	14 (31.8%)	< 0.001
*Respiratory support at 40 weeks PMA*					< 0.001
None	30 (100%)	31 (100%)	18 (78.3%)	19 (43.2%)	
Oxygen or flow	0 (0)	0 (0)	2 (8.7%)	19 (43.2%)	
MV or deceased	0 (0)	0 (0)	3 (13.0%)	6 (13.6%)	
*Oxygen requirement at discharge*					0.009
None	30 (100%)	31 (100%)	20 (87.0%)	36 (81.8%)	
Oxygen or deceased	0 (0)	0 (0)	3 (13.0%)	8 (18.2%)	
Duration of mechanical ventilation, days	0 (0, 0)	1 (0, 7)	9 (2, 21)	13 (7, 32)	0.004
Duration of supplemental oxygen, days	0 (0, 7)	48 (34, 57)	71 (48, 87)	98 (73, 120)	0.30
Length of hospital stay, days	36 (19, 44)	75 (63, 80)	87 (68, 110)	111 (94, 127)	< 0.001

BPD, bronchopulmonary dysplasia; Non-BPD, non-bronchopulmonary dysplasia; PMA, postmenstrual age; GA, gestational age; IVH, intraventricular hemorrhage; NEC, necrotizing enterocolitis; ROP, retinopathy of prematurity

The mLUS score for the whole cohort was 14 (IQR 8–17), subjects with BPD was 16 (IQR 8–17) and subjects without BPD was 3 (IQR 1–6). Inter-observer variability for mLUS score shows good consistency. The measured variability (intra-subject coefficient of variation) for inter-observer differences was 3.0%.

**Fig. 2:**
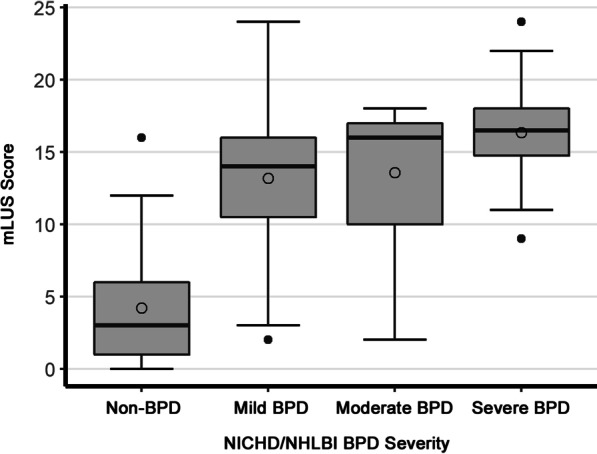
Correlation between mLUS score and NICHD/NHLBI BPD severity. The mLUS score is significantly correlated with NICHD/NHLBI BPD severity adjusted for gestational age (*P* < 0.001). Plot elements are represented as follows: mean (circle); median (horizontal line); interquartile range (gray box); and 95% confidence interval (whiskers). BPD: bronchopulmonary dysplasia; mLUS score: modified lung ultrasound score

**Fig. 3:**
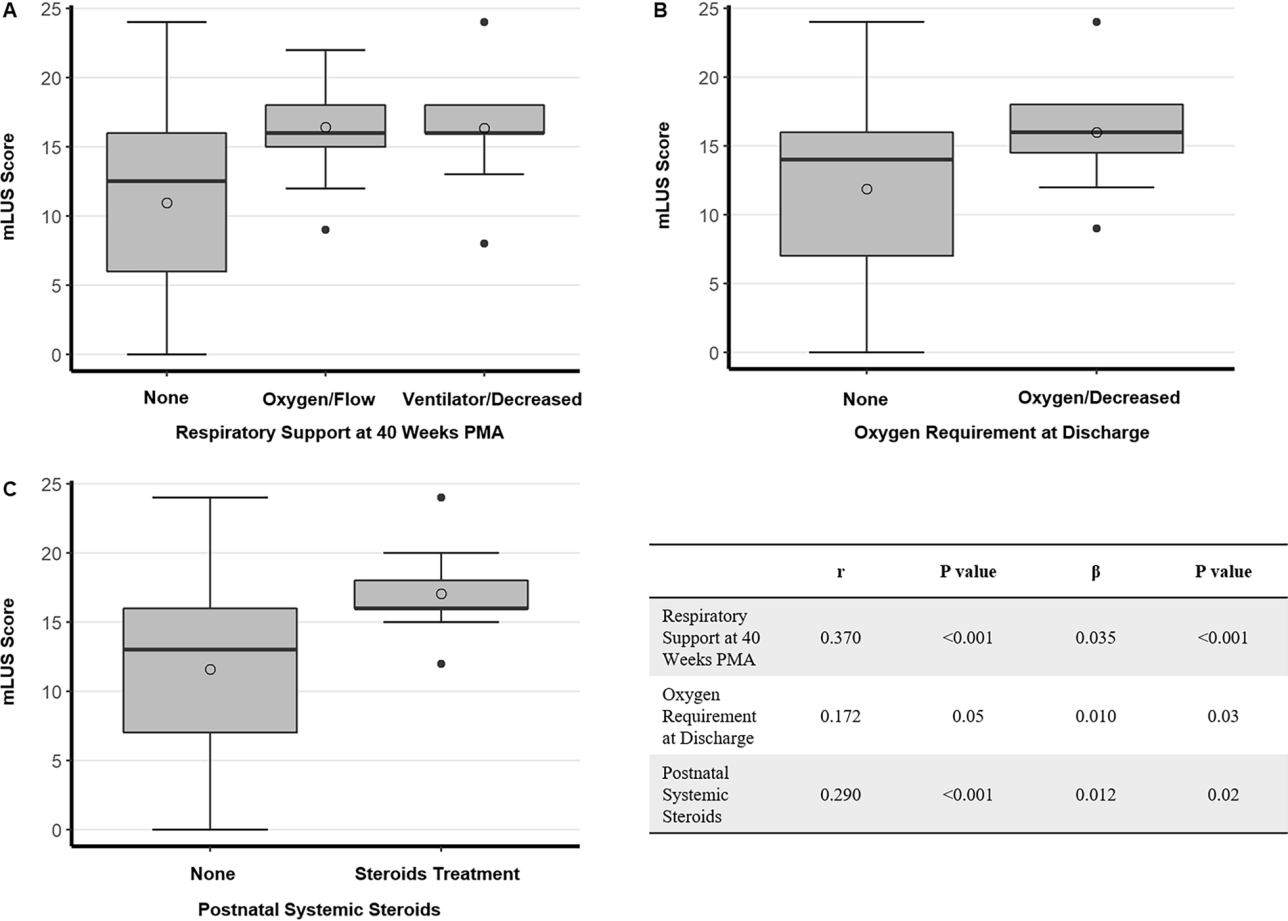
Correlation between mLUS score and short-term outcomes described as categorical variables. Panel **A**, **B** and **C** represent respiratory support at 40 weeks PMA, oxygen requirement at discharge and postnatal systemic steroids, respectively. The mLUS score is significantly correlated with respiratory support at 40 weeks PMA (*P* < 0.001), oxygen requirement at discharge (*P* = 0.03) and postnatal systemic steroids (*P* = 0.02) adjusted for gestational age. Plot elements are represented as follows: mean (circle); median (horizontal line); interquartile range (gray box); and 95% confidence interval (whiskers). Table shows Spearman correlation coefficients and results of multivariate linear regressions adjusted for gestational age. mLUS score: modified lung ultrasound score; r: Spearman correlation coefficient; β: adjusted linear regression coefficient

**Fig. 4:**
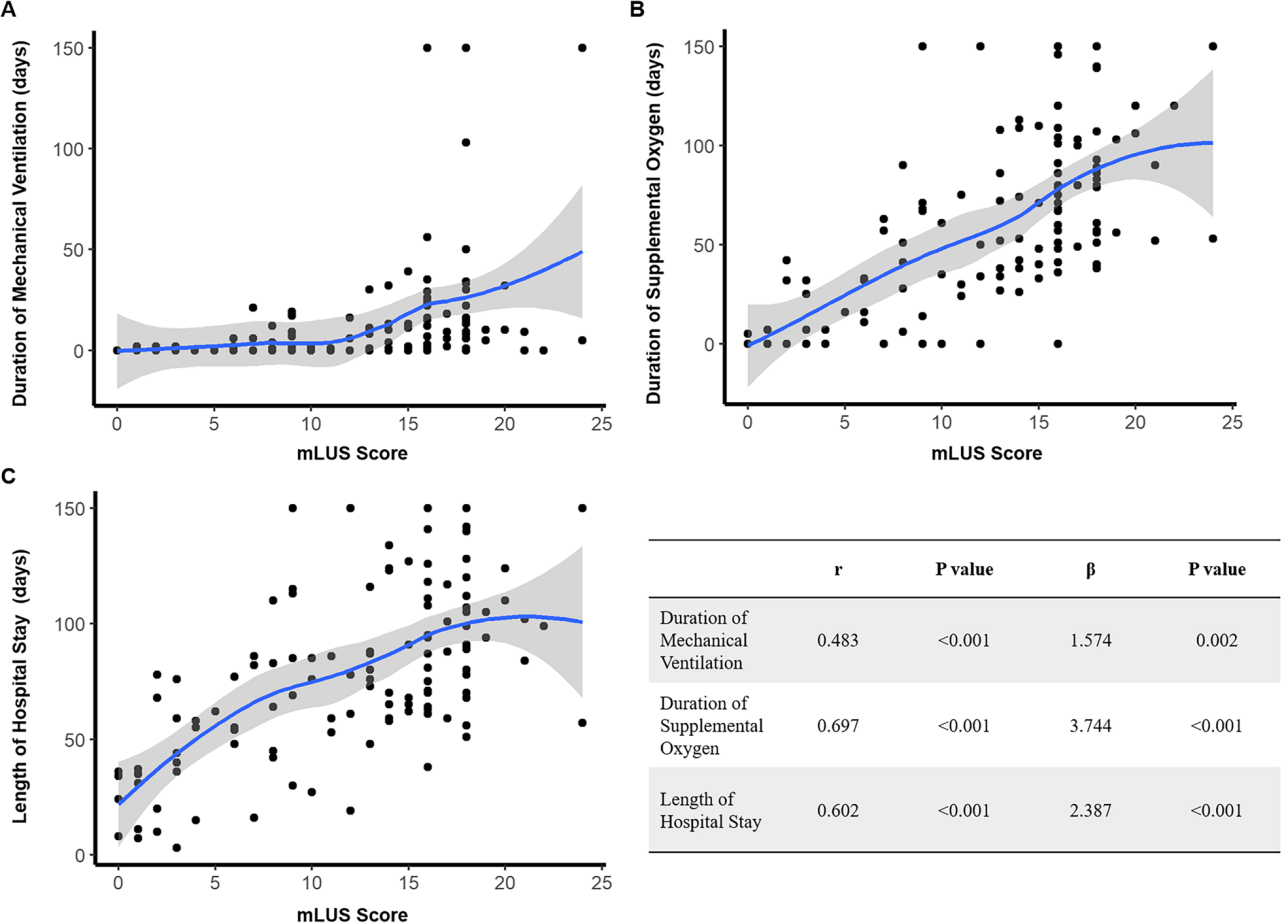
Correlation between mLUS score and short-term outcomes described as continuous variables. Panel **A**, **B** and **C** represent duration of mechanical ventilation, duration of supplemental oxygen and length of hospital stay, respectively. The mLUS score is significantly correlated with duration of mechanical ventilation (*P* = 0.002), duration of supplemental oxygen (*P* < 0.001) and length of hospital stay (*P* < 0.001) adjusted for gestational age. Hatched blue curves represent the best fitting data lines and are all generated by local regression smoothing procedure. Table shows Spearman correlation coefficients and results of multivariate linear regressions adjusted for gestational age. mLUS score: modified lung ultrasound score; r: Spearman correlation coefficient; β: adjusted linear regression coefficient

Although both mLUS score and cLUS score were significantly correlated with NICHD/NHLBI BPD severity, the superiority of mLUS score was greater for concrete BPD severity (Fig. 2 and Additional file 1: Figure S2). The mLUS score significantly correlated with all the short-term clinical outcomes adjusted for gestational age (Figs. 3 and 4). The cLUS scores were also associated with most outcomes, except for oxygen requirement at discharge and postnatal systemic steroids treatment, but the correlation was weaker than mLUS scores (See Additional file 1: Figures S3 and S4).

**Fig. 5:**
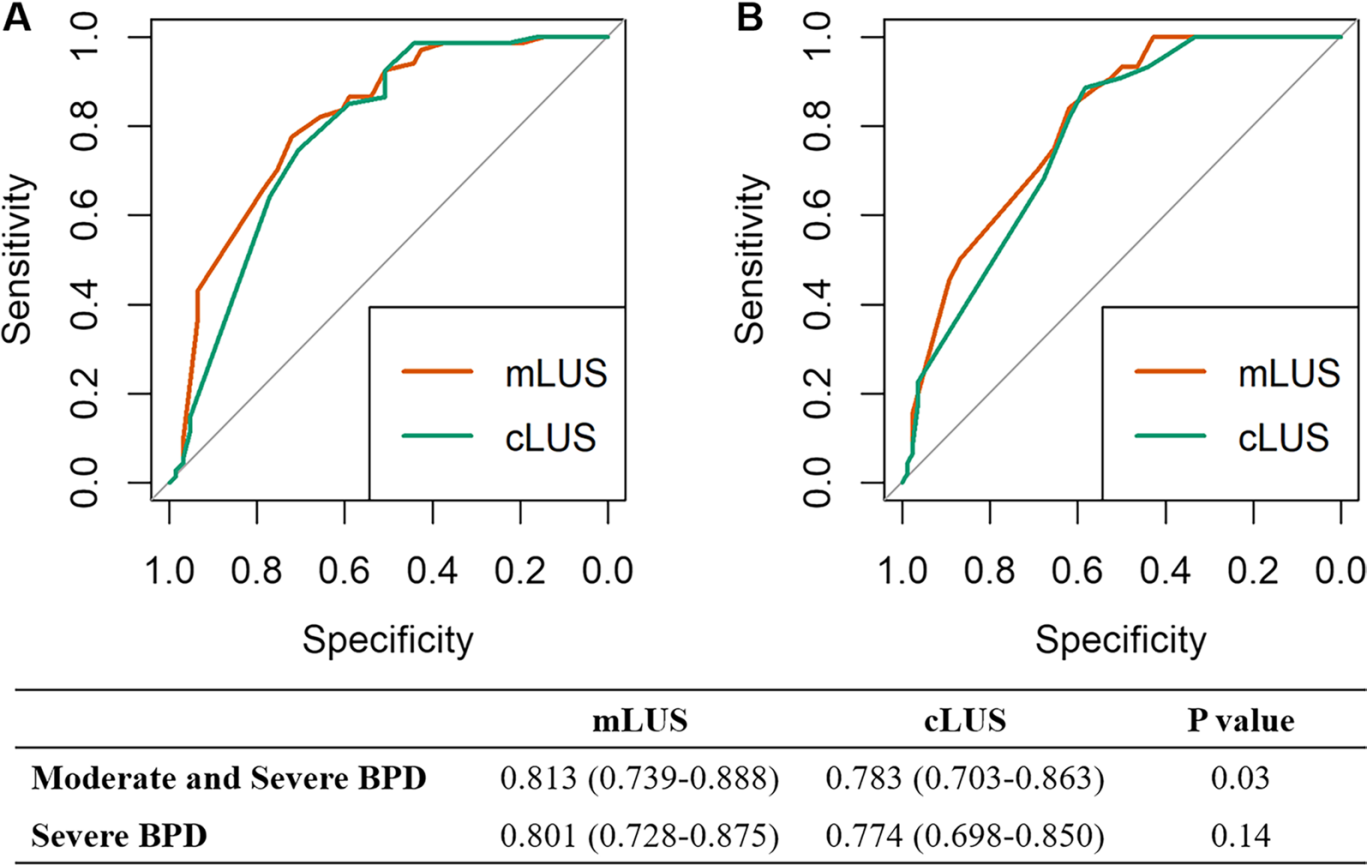
Receiver operating characteristic curves for assessing NICHD/NHLBI BPD severity using mLUS score and mLUS score. Two Panels respectively represent ROC curves for **A** moderate and severe BPD and **B** severe BPD. Different colors represent ROC curves for mLUS score (red) and cLUS score (green). Grey line represents the reference line. The areas under the curves for moderate and severe BPD shows significant difference between mLUS score and cLUS score. Table shows areas under the curves and 95% confidence interval. *P*-values for these comparisons are calculated. BPD: bronchopulmonary dysplasia; ROC curve: receiver operating characteristic curve; mLUS score: modified lung ultrasound score; cLUS score: classic lung ultrasound score

Figure 5A showed the ROC analysis for mLUS score and cLUS score in differentiating subjects with moderate and severe BPD: AUC = 0.813 (95% CI 0.739–0.888) for mLUS score and AUC = 0.783 (95% CI 0.703–0.863) for cLUS score, which were significantly different (P = 0.03). Figure 5B showed the ROC analysis to differentiate subjects with severe BPD: AUC = 0.801 (95% CI 0.728–0.875) for mLUS score and AUC = 0.774 (95% CI 0.698–0.850) for cLUS score. The AUC for mLUS score was higher than that for cLUS score, but the difference was not significant. ROC analyses evaluating other short-term clinical outcomes also showed significant differences between mLUS and cLUS scores (See Additional file 1: Figure S5). Table 2 showed the optimal cutoff point for the mLUS score to differentiate subjects with moderate and severe BPD/severe BPD. The optimal cutoff points for mLUS score were 14 with the highest Youden’s index in the ROC analysis for moderate and severe BPD and 16 for severe BPD. Stratification of participants according to mLUS score < 14 vs mLUS score ≥ 14 showed significant differences in short-term clinical outcomes except for oxygen requirement at discharge either adjusted for gestational age or not (See Additional file 1: Table S2). The optimal cutoff points for the cLUS score and comparison between stratifications were also shown in Additional file 1: Table S3 and S4.

**Table 2:** Reliability of mLUS Score for NICHD/NHLBI BPD Severity

	Best Cutoff Value	Sensitivity (%)	Specificity (%)	Positive LR	Negative LR	PPV (%)	NPV (%)	Posttest Probability (%)
Moderate and Severe BPD	≥ 14	77.6	72.1	2.78	0.31	75.4	74.6	75.0
Severe BPD	≥ 16	70.5	69.1	2.28	0.43	54.4	81.7	69.5

BPD, bronchopulmonary dysplasia; LR, likelihood ratio; NPV, negative predictive value; PPV, positive predictive value

## Discussion

In this study, we evaluated BPD in preterm infants with GA < 34 weeks by lung ultrasound. We herein propose a modified semi-quantitative score based on lung ultrasound to estimate BPD severity. Our results show that: (1) mLUS score significantly correlates with short-term clinical outcomes of BPD; (2) mLUS scores have better reliability for short-term clinical outcomes compared with cLUS score. These findings suggest that lung ultrasound can accurately and noninvasively assess the severity of BPD at 36 weeks of PMA and evaluate short-term clinical outcomes. In addition, these results suggest that the modified semi-quantitative lung ultrasound score can play an important and possibly crucial role in assessing the severity of BPD, which potentially meets requirements in management of BPD infants.

The concept of BPD was first proposed by Northway in 1967, based on radiological and pathological examination of neonates with severe chronic lung injury [1]. Current NICHD/NHLBI BPD definitions of supplemental oxygen requirements at a single time point cannot cover all infants with chronic respiratory insufficiency associated with preterm birth [3, 30]. In addition, the non-uniform criteria for oxygen use in infants with respiratory diseases among centers or clinicians interfere diagnosis of BPD [31]. These problems in the definition of BPD increase the difficulties of designing clinical trials for drug development and therapeutic advance, even more difficulties in inter-center comparisons. A systematic review found that many clinical trials did not use a discrete definition of BPD [32]. This situation is complicated by the lack of imaging techniques to characterize BPD [33]. In 2017, the importance of imaging to characterize BPD was recognized at Bronchopulmonary Dysplasia Workshop conference sponsored by the National Institutes of Health. The workshop consensus suggested adding radiographic confirmation to the definition of BPD. However, current modalities in BPD diagnoses such as serial chest X-ray, CT-scan have either poor correlation with BPD severity and clinical outcomes or concerning regarding high radiation exposure [34, 35]. Pulmonary MRI can describe the severity of lung disease without ionizing radiation, but is not always available in an intensive care unit setting [36]. Lung ultrasound, as is radiation-free, portable, noninvasive and available to most NICUs even in developing countries, may be a potential modality in confirming BPD definition.

Our results are consistent with those of previous studies. These studies focused on predicting the occurrence of BPD within 28 days after birth [14–19]. Early prediction of BPD occurrence is essential to proactively take measures to decrease the risk of BPD, such as postnatal steroids. However, these studies did not characterize the lung ultrasound imaging of BPD at 36 weeks PMA. Thirty-six weeks PMA is an important time-point to evaluate lung lesions severity and prognosis of preterm infants. As above, current NICHD/NHLBI BPD definitions of exclusive supplemental oxygen requirements cannot well reflect disease phenotype and lung lesion severity. Therefore, lung ultrasound imaging is potential to be part of a more global definition and severity criterion of BPD.

To the best of our knowledge, we prospectively use lung ultrasound scores in a relatively large-sized population for the first time to evaluate BPD severity at 36 weeks PMA timepoint and study the correlation of LUS scores with short-term clinical outcomes. In addition, we have innovatively modified the lung ultrasound scoring system by integrating classic six-chest area ultrasound with lung base ultrasound through the liver and spleen. In BPD patients, the lung lesions of BPD patients may be physiologically worse in posterior (beside vertebra) and lower parts (close to diaphragm) than anterior areas. Thus, cLUS score without assessing the severe parts of the lung potentially need to be improved. As such, we generated a modified mLUS by integrating retrodiaphragmatic lung ultrasound images with cLUS. Additionally, since liver and spleen provide perfect ultrasound window, retrodigphragmtic lung ultrasound images are easy to access. In this prospective study, we demonstrated that mLUS score could more accurately assist BPD diagnosis and had better reliability for predicting short-term clinical outcomes compared with cLUS score. This provides a more reliable imaging tool. Thus, it is possible to personalize the assessment of infants who develop BPD on a certain scale.

The mLUS score is well correlated with NICHD/NHLBI BPD severity and the other short-term clinical outcomes. Even though gestational age plays an evident role in the development of BPD, our results are still consistently significant after adjustment for gestational age. This shows mLUS score may be able to describe BPD severity independent from gestational age. Of note, the correlation with oxygen requirement at discharge is significant for mLUS score but not for cLUS score. And ROC analysis of mLUS score to evaluate short-term outcomes such as BPD severity and oxygen requirement at discharge also showed significant superiority to cLUS score. Therefore, mLUS score shows better diagnostic performance in differentiating BPD severity and evaluating the other short-term clinical outcomes than cLUS score. This indicates the participation of lung base ultrasound through liver and spleen has significantly improved the reliability of lung ultrasound score system. In contrast to the previous result of no improved reliability investigated when dividing the chest into more areas [16], our results show significantly improved reliability. This is likely in that lung base aeration is better correlated with lung disease severity than posterior lung areas in infants with BPD. However, our study was not designed to compare lung base areas and posterior lung areas, although interesting, needs to be further investigated. In addition, mLUS score above 14 is significantly correlated with worse outcomes, independent from gestational age. The cut-off values of mLUS may also represent an easy tool to estimate the BPD severity and guide further examinations and interventions.

The main strength of our study is that it is a prospective study based on a previous design. Enough participants were recruited to examine the reliability of the mLUS score in predicting short-term clinical outcomes. We have taken as comprehensive measures as possible to give blinding to investigators and all the participants received standardized respiratory management based on formal protocols to reduce the risk of bias. We have creatively integrated classic six-chest area ultrasound and lung base ultrasound to significantly improve the reliability of evaluating short-term clinical outcomes. This fills a gap in assessing the severity of the disease in infants with BPD using simple, rapid, and radiation-free imaging tools.

We acknowledge some research limitations. More recent BPD definitions and severity criteria such as physiological definition and Jensen definition were not used in our study because they were not available in our NICU at the outset [37, 38]. However, the modified NICHD/NHLBI definition we use is widely accepted. We believe that our result may be revelatory to integrate lung ultrasound into more accurate BPD definitions and severity criteria. Pulmonary vascular disease and large airway disease are also important components of severe BPD that can’t be evaluated with lung ultrasound [39]. No arterial or percutaneous blood gas analysis was performed during lung ultrasonography to investigate the correlation between mLUS scores and lung aeration. However, this has been confirmed in previous studies [11, 16]. Ultrasound is usually thought of as operator-dependent. However, our data indicate a high level of agreement among observers, which is consistent with results of previous studies [11, 15]. In addition, the lung ultrasound learning curve is quite short, and different ultrasonic probes may not affect the interpretation of basic lung ultrasound results [40]. Our mLUS score system required two different probes and more manipulation, which implied more time consuming to the operator and more uncomfortable to infants, though better reliability has been confirmed. Since retrodigphragmtic lung ultrasound images are easy to access, adding two more images doesn’t really add too many technique difficulties to ultrasound operators. Finally, the short-term clinical outcomes were censored before discharge in this study. Further longitudinal studies are needed to investigate the relationship between lung ultrasound imaging and long-term outcomes.

## Conclusions

The mLUS score at 36 weeks PMA correlates significantly with short-term clinical outcomes in preterm infants. These results can be used to evaluate the BPD severity of preterm infants and promote the improvement of current BPD definitions.

## Supplementary Information

**Additional file 1**. **Appendix 1**. Standardized Respiratory Care and BPD Procedures; **Appendix 2**. Lung Ultrasound Protocol; **Table S1**. Modified NICHD/NHLBI Definition of BPD with Severity; **Table S2**. Frequency Distribution of Short-Term Clinical Outcomes Across mLUS Score; **Table S3**. Reliability of cLUS Score for NICHD/NHLBI BPD Severity; **Table S4**. Frequency Distribution of Short-Term Clinical Outcomes Across cLUS Score; **Figure S1**. Flow Chart of The Study Population; **Figure S2**. Correlation Between cLUS Score and NICHD/NHLBI BPD Severity; **Figure S3**. Correlation Between cLUS Score and Short-Term Outcomes Described as Categorical Variable; **Figure S4**. Correlation Between cLUS Score and Short-term Outcomes Described as Continuous Variable; **Figure S5**. Receiver Operating Characteristic Curves for Evaluating Short-Term Clinical Outcomes Using mLUS Score and mLUS Score.

## Data Availability

The datasets used and analyzed during the current study available from the corresponding authors on reasonable request.

## Abbreviations

AUC: Area under the curve
BPD: Bronchopulmonary dysplasia
cLUS score: Classic lung ultrasound score
GA: Gestational age
IQR: Interquartile range
LUS: Lung ultrasound
mLUS score: Modified lung ultrasound score
NICHD: National Institute of Child Health and Human Development
NICU: Neonatal intensive care unit
PMA: Postmenstrual age
ROC: Receiver operating characteristic
