# Supplementary material for: A modified lung ultrasound score to evaluate short-term clinical outcomes of bronchopulmonary dysplasia
Source: BMC Pulm Med. 2022 Mar 19;22:95. doi: 10.1186/s12890-022-01885-4 (PMC8933905; doi:10.1186/s12890-022-01885-4)
Supplement: Supplementary file 1 — Additional file 1. Appendix 1. Standardized Respiratory Care and BPD Procedures; Appendix 2. Lung Ultrasound Protocol; Table S1. Modified NICHD/NHLBI Definition of BPD with Severity; Table S2. Frequency Distribution of Short-Term Clinical Outcomes Across mLUS Score; Table S3. Reliability of cLUS Score for NICHD/NHLBI BPD Severity; Table S4. Frequency Distribution of Short-Term Clinical Outcomes Across cLUS Score; Figure S1. Flow Chart of The Study Population; Figure S2. Correlation Between cLUS Score and NICHD/NHLBI BPD Severity; Figure S3. Correlation Between cLUS Score and Short-Term Outcomes Described as Categorical Variable; Figure S4. Correlation Between cLUS Score and Short-term Outcomes Described as Continuous Variable; Figure S5. Receiver Operating Characteristic Curves for Evaluating Short-Term Clinical Outcomes Using mLUS Score and mLUS Score. [file 12890_2022_1885_MOESM1_ESM.pdf]

Additional Files

A Modified Lung Ultrasound Score to Evaluate Short-Term Clinical Outcomes of Bronchopulmonary Dysplasia

Appendix 1. Standardized Respiratory Care and BPD Procedures

Appendix 2. Lung Ultrasound Protocol

Table S1. Modified NICHD/NHLBI Definition of BPD with Severity

Table S2. Frequency Distribution of Short-Term Clinical Outcomes Across mLUS Score

Table S3. Reliability of cLUS Score for NICHD/NHLBI BPD Severity

Table S4. Frequency Distribution of Short-Term Clinical Outcomes Across cLUS Score

Figure S1. Flow Chart of The Study Population

Figure S2. Correlation Between cLUS Score and NICHD/NHLBI BPD Severity

Figure S3. Correlation Between cLUS Score and Short-Term Outcomes Described as Categorical Variable

Figure S4. Correlation Between cLUS Score and Short-term Outcomes Described as Continuous Variable

Figure S5. Receiver Operating Characteristic Curves for Evaluating Short-Term Clinical Outcomes Using mLUS Score and mLUS Score.

Appendix 1. Standardized Respiratory Care and BPD Procedures

Domain	Comments
Threatened preterm labour	Encourage obstetrician to administer prenatal steroids to women at risk for preterm labor < 34 weeks gestation
Delivery Room – CPAP or T-piece with PEEP	CPAP 5- 8cmH_2O<br>Initiate using room air (or 30% O_2 if gestational age < 28weeks)<br>Titrate O_2 up as necessary to achieve PREDUCTAL saturation of >80% by 5 minutes of age<br>Consider intubation if requiring FiO_2<br>>30-40% and increasing after initial transitioning
Early use of surfactant	If baby requires mechanical ventilation for infant respiratory distress syndrome<br>If oxygen requirement becomes greater than 30% on NCPAP/NIPPV
NIPPV/CPAP	Maintain consistent NCPAP especially if under 28 weeks gestation<br>If not stable, change to NIPPV, PIP/PEEP 14-18/6-8cmH_2O, Ti 0.5-1.0s, f 30-45/min<br>Maintain baby on CPAP/NIPPV for at least one week if under 29 weeks gestation
Mechanical Ventilation	Aim to avoid mechanical ventilation<br>If mechanical ventilation is necessary, use all tools available to minimize ventilator induced lung injury (VILI)<br>Permissive hypercapnia, PCO2 45-55mmHg, pH>7.22
Ventilatory Mode	Prevent volutrauma and barotrauma (preference is volume targeted) Volume Guarantee on the VN-500 (VG 4.0-6.0ml/kg<br>Use HFO+VG as second choice, HFO VG 1.5-2.2ml/kg
Avoid lung derecruitment	Use in line suction<br>Avoid ventilator disconnects

Extubation	Extubate as early as possible<br>Always extubate to distending pressure (prefer NIPPV).<br>Do not use high flow nasal prongs if gestational age <28weeks
Oxygen Therapy	Always use blenders when on oxygen<br>Target O_2 Saturation 91-94%<br>High alarm set as 95% when on O_2
Medical Therapy	Early Caffeine citrate, bolus dose 20mg/kg, then 10mg/kg.d until 33-34weeks PMA.<br>DART (DEX cumulative dose of 0.89mg for 10days) protocol for patients on mechanical ventilation >10-14 days and severe lung interstitial lesions, exclude CMV/UU infection beforehand.<br>Avoid high fluid intake.

Appendix 2. Lung Ultrasound Protocol.

Each lung has been divided into 4 areas (upper anterior, lower anterior, lateral and retrodiaphragmatic lung base areas). Transversal and longitudinal scans of the anterior and lateral chest walls were performed to detect upper anterior, lower anterior, lateral lung areas with a linear probe and sagittal scans were performed through the right lobe of the liver and through the mid-spleen to detect retrodiaphragmatic lung base appearance with a convex probe.

The classic lung ultrasound (cLUS) score was calculated considering 6 chest areas, as previous studies described. The modified lung ultrasound (mLUS) score was calculated by including the retrodiaphragmatic lung base areas, thus considering 8 chest areas. For each area, a 0- to 3-score was given, total score ranging from 0 to 24. Score values correspond to the different lung ultrasound semiology patterns, as shown in the middle and lower parts of the Figure 1.

The patterns for linear probe are scored as previously described: 0 indicates A-pattern (defined as the presence of only A-lines); 1, B-pattern (defined as the presence of isolated B-lines, well-spaced); 2, severe B-pattern (defined as the presence of crowded and coalescent B lines); and 3, consolidations pattern (defined as the presence of extended consolidations). Scores are given for convex probe as follows: 0 indicates normal pattern (defined as the presence of only normal retrodiaphragmatic hypoechogenicity); 1, initial pattern (defined as the presence of isolated, well-spaced retrodiaphragmatic hyperechogenicity); 2, severe hyperechogenicity pattern (defined as the presence of crowded and coalescent hyperechogenicity without consolidations); 3, diffuse hyperechogenicity pattern (defined as the presence of diffuse hyperechogenicity over the whole area) or consolidations pattern (defined as the presence of extended consolidations).

Table S1. Modified NICHD/NHLBI Definition of BPD with Severity

BPD is defined as requiring oxygen support for at least 28 days. The severity was classified by oxygen requirement at 36 weeks PMA in infants <32 weeks' GA and at 56 days of life in infants with older GA.

BPD severity	Definition
None BPD	O_2 demand < 28 days
Mild BPD	O_2 demand at least 28 days and breathing room air at 36 weeks PMA (GA < 32 weeks) or 56 days of life (GA ≥ 32 weeks)
Moderate BPD	O_2 demand at least 28 days and oxygen requirements < 30% at 36 weeks PMA (GA < 32 weeks) or 56 days of life (GA ≥ 32 weeks)
Severe BPD	O_2 demand at least 28 days and HFNC/CPAP/PPV and/or oxygen requirement ≥ 30% at 36 weeks PMA (GA < 32 weeks) or 56 days of life (GA ≥ 32 weeks)

O2: oxygen; HFNC: high flow nasal cannula; CPAP: continuous positive pressure; IPPV: positive pressure ventilation.

Table S2. Frequency Distribution of Short-Term Clinical Outcomes Across mLUS Score

Outcome	mLUS Score < 14	mLUS Score ≥ 14	OR (95% CI)	P value	Adjust OR (95% CI)	Adjust P value
Moderate and severe BPD	15 (25.4%)	52 (75.4%)	8.97 (4.02, 20.01)	< 0.001	6.18 (2.65-14.39)	< 0.001
Oxygen requirement or deceased at 40 weeks PMA	5 (8.5%)	25 (36.2%)	6.14 (2.17, 17.35)	0.001	5.10 (1.69, 15.39)	0.004
Oxygen requirement or deceased at discharge	3 (5.1%)	8 (11.6%)	2.45 (0.62, 9.69)	0.20	2.53 (0.56, 11.43)	0.23
Postnatal systemic steroids treatment	1 (1.7%)	14 (20.3%)	14.76 (1.88, 116.1)	0.01	9.79 (1.18, 80.93)	0.03
Duration of mechanical ventilation > 14 days	5 (8.5%)	27 (39.1%)	6.94 (2.46, 19.56)	< 0.001	4.79 (1.61, 14.22)	0.005
Duration of supplemental oxygen > 60 days	12 (20.3%)	45 (65.2%)	7.34 (3.28, 16.42)	< 0.001	4.34 (1.81, 10.42)	0.001
Length of hospital stay > 90 days	6 (10.2%)	38 (55.1%)	10.83 (4.11, 28.52)	< 0.001	6.91 (2.51, 19.02)	< 0.001

Data are presented as n (%). mLUS score: modified lung ultrasound score; BPD: bronchopulmonary dysplasia; OR: odd ratio; CI: confidence interval.

Table S3. Reliability of cLUS Score for NICHD/NHLBI BPD Severity

	Best Cutoff Value	Sensitivity, %	Specificity, %	Positive LR	Negative LR	PPV	NPV	Posttest Probability, %
Moderate and Severe BPD	≥ 11	74.6%	70.5%	2.53	0.36	73.5%	71.7%	72.7%
Severe BPD	≥ 13	22.7%	96.4%	6.36	0.80	76.9%	70.4%	71.1%

BPD, bronchopulmonary dysplasia; LR, likelihood ratio; NPV, negative predictive value; PPV, positive predictive value.

Table S4. Frequency Distribution of Short-Term Clinical Outcomes Across cLUS Score

Outcome	cLUS Score < 11	cLUS Score ≥ 11	OR (95% CI)	P value	Adjust OR (95% CI)	Adjust P value
Moderate and severe BPD	17 (28.3%)	50 (73.5%)	7.03 (3.23, 15.30)	< 0.001	4.79 (2.10, 10.93)	< 0.001
Oxygen requirement or deceased at 40 weeks PMA	7 (11.7%)	23 (33.8%)	3.87 (1.52, 9.85)	0.005	3.03 (1.13, 8.18)	0.03
Oxygen requirement or deceased at discharge	5 (8.3%)	6 (8.8%)	1.06 (0.31, 3.68)	0.92	0.93 (0.24, 3.60)	0.92
Postnatal systemic steroids treatment	3 (5.0%)	12 (17.6%)	4.07 (1.09, 15.21)	0.04	2.55 (0.65, 10.09)	0.18
Duration of mechanical ventilation > 14 days	8 (13.3%)	24 (35.3%)	3.55 (1.45, 8.68)	0.006	2.32 (0.90, 6.02)	0.08
Duration of supplemental oxygen > 60 days	13 (21.7%)	44 (64.7%)	6.63 (3.01, 14.61)	< 0.001	4.03 (1.70, 9.59)	0.002
Length of hospital stay > 90 days	9 (15.0%)	35 (51.5%)	6.01 (2.56, 14.11)	< 0.001	3.76 (1.51, 9.35)	0.004

Data are presented as n (%). cLUS score: classic lung ultrasound score; BPD: bronchopulmonary dysplasia; OR: odd ratio; CI: confidence interval.

Figure S1. Flow Chart of The Study Population.

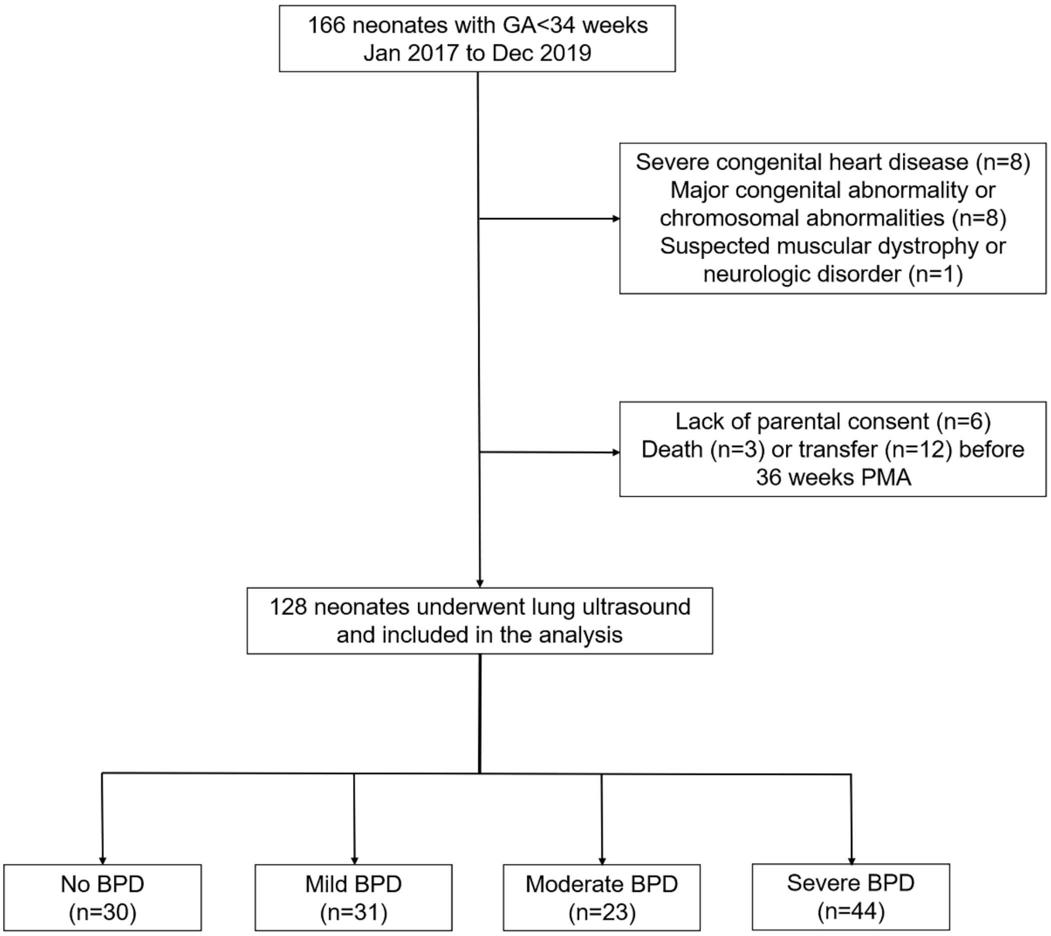

GA: gestational age; BPD: bronchopulmonary dysplasia.

Figure S2. Correlation between cLUS score and NICHD/NHLBI BPD severity.

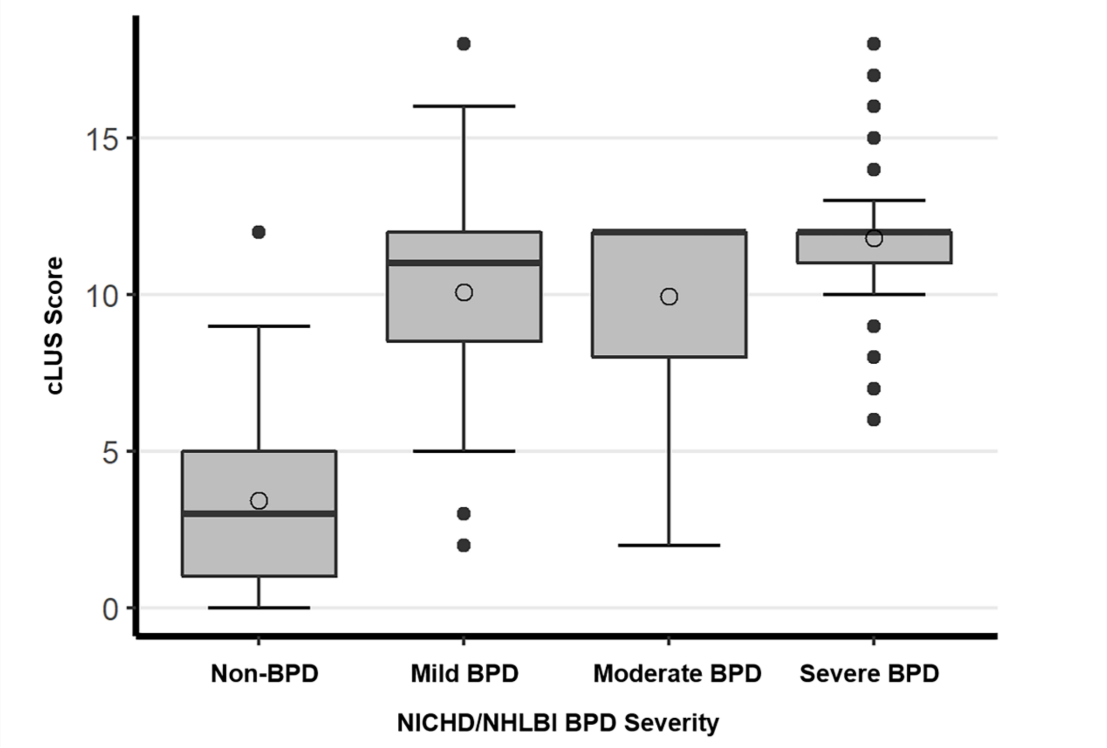

The cLUS score is significantly correlated with NICHD/NHLBI BPD severity adjusted for gestational age ($P < 0.001$). Plot elements are represented as follows: mean (circle); median (horizontal line); interquartile range (gray box); and 95% confidence interval (whiskers). BPD: bronchopulmonary dysplasia; cLUS score: classic lung ultrasound score.

Figure S3. Correlation Between cLUS Score and Short-Term Outcomes Described as Categorical Variable.

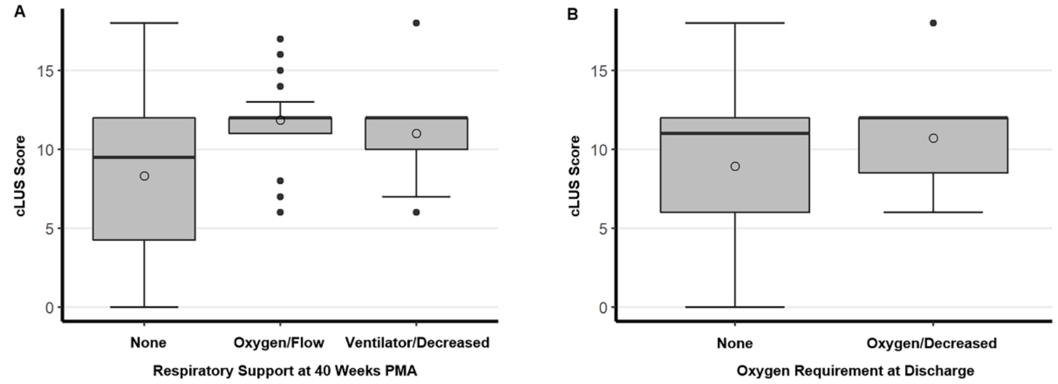

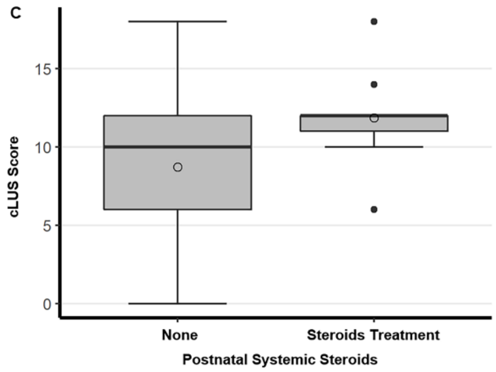

	r	P value	β	P value
Respiratory Support at 40 Weeks PMA	0.300	<0.001	0.036	0.008
Oxygen Requirement at Discharge	0.085	0.34	0.008	0.22
Postnatal Systemic Steroids	0.224	0.01	0.012	0.12

Panel A, B and C represent respiratory support at 40 weeks PMA, oxygen requirement at discharge and postnatal systemic steroids, respectively. The cLUS score is only significantly correlated with respiratory support at 40 weeks PMA (P = 0.008), but not significant for oxygen requirement at discharge (P = 0.22) and postnatal systemic steroids (P = 0.12) adjusted for gestational age. Plot elements are represented as follows: mean (circle); median (horizontal line); interquartile range (gray box); and 95% confidence interval (whiskers). Table shows Spearman correlation coefficients and results of multivariate linear regressions adjusted for gestational age. cLUS score: classic lung ultrasound score; r: Spearman correlation coefficient; β: adjusted linear regression coefficient.

Figure S4. Correlation Between cLUS Score and Short-term Outcomes Described as Continuous Variable.

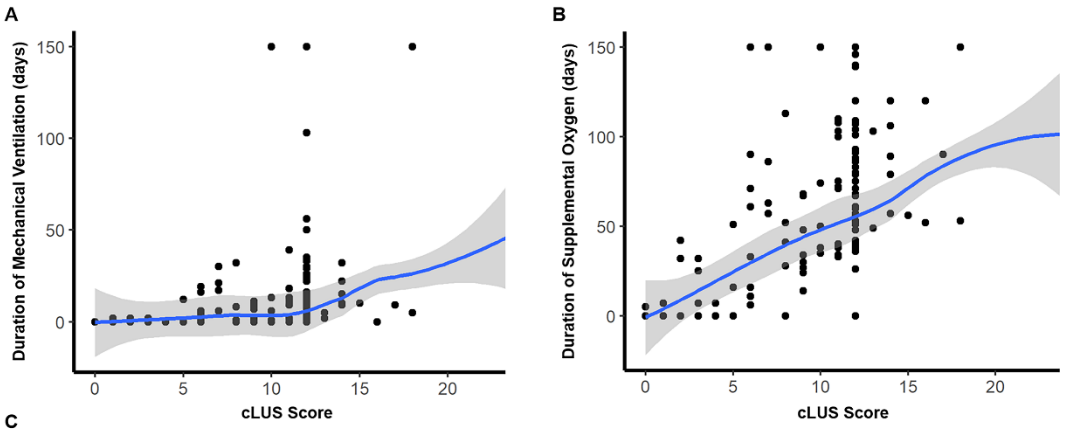

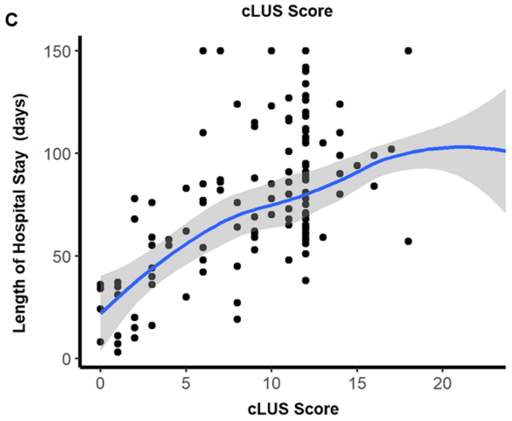

	r	P value	β	P value
Duration of Mechanical Ventilation	0.428	<0.001	1.565	0.03
Duration of Supplemental Oxygen	0.638	<0.001	4.526	<0.001
Length of Hospital Stay	0.557	<0.001	3.176	<0.001

Panel A, B and C represent duration of mechanical ventilation, duration of supplemental oxygen and length of hospital stay, respectively. The cLUS score is significantly correlated with duration of mechanical ventilation (P=0.03), duration of supplemental oxygen (P<0.001) and length of hospital stay (P<0.001) adjusted for gestational age. Hatched blue curves represent the best fitting data lines and are all generated by local regression smoothing procedure. Table shows Spearman correlation coefficients and results of multivariate linear regressions adjusted for gestational age. cLUS score: classic lung ultrasound score; r: Spearman correlation coefficient; β: adjusted linear regression coefficient.

Figure S5. Receiver Operating Characteristic Curves for Evaluating Short-Term Clinical Outcomes Using mLUS Score and mLUS Score.

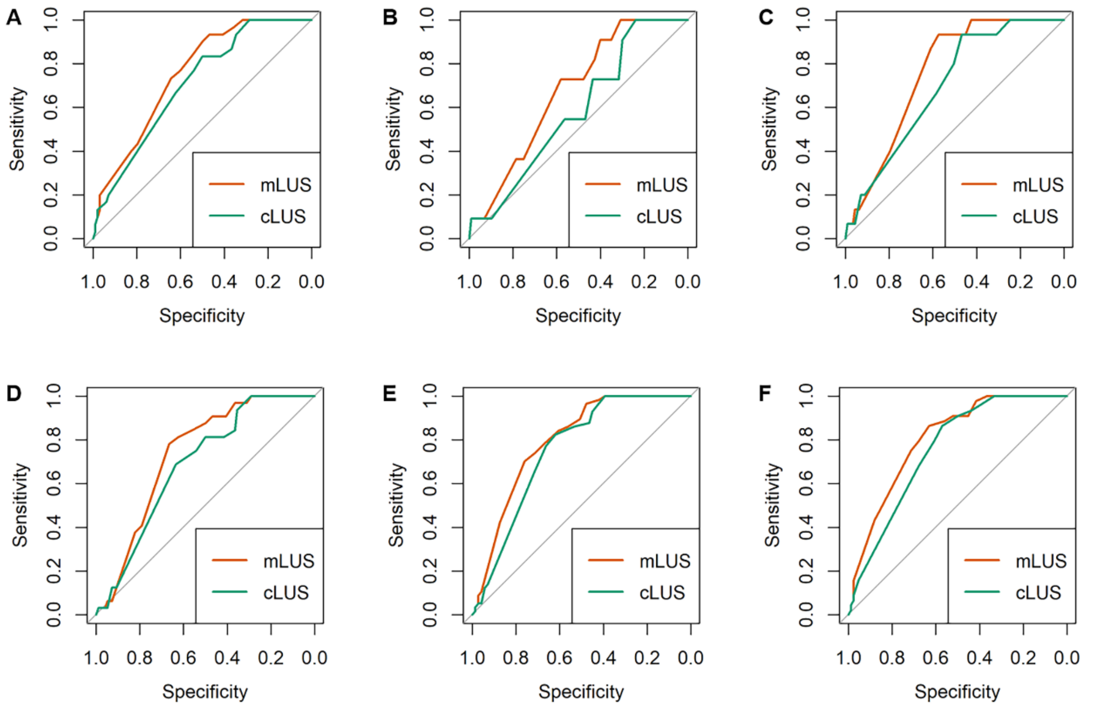

	mLUS	cLUS	P value
**Oxygen requirement at 40 weeks PMA**	0.753 (0.666-0.840)	0.708 (0.614-0.801)	0.04
**Oxygen requirement at discharge**	0.676 (0.539-0.813)	0.586 (0.434-0.737)	0.006
**Postnatal systemic steroids treatment**	0.759 (0.665-0.853)	0.697 (0.583-0.811)	0.04
**Duration of mechanical ventilation > 14 days**	0.737 (0.651-0.823)	0.688 (0.597-0.779)	0.02
**Duration of supplemental oxygen > 60 days**	0.796 (0.720-0.872)	0.756 (0.674-0.837)	0.02
**Length of hospital stay > 90 days**	0.804 (0.730-0.878)	0.756 (0.678-0.835)	0.01

Panels A to F represent ROC curves for (A) oxygen support at 40 weeks PMA; (B) oxygen requirement at discharge; (C) postnatal systemic steroids treatment; (D) duration of mechanical ventilation > 14 days; (E) duration of supplemental oxygen > 60 days; and (F) length of hospital stay > 90 days, respectively. Different colors represent ROC curves for mLUS score (red) and cLUS score (green). Grey line represents the reference line. The areas under the curves for all the outcomes show significantly difference between mLUS score and cLUS score. Table shows areas under the curves and 95% confidence interval. P-values for these comparisons are calculated. ROC curve: receiver operating characteristic curve; mLUS score: modified lung ultrasound score; cLUS score: classic lung ultrasound score; PMA: postmenstrual age.
